# The *Vibrio*-Squid Symbiosis as a Model for Studying Interbacterial Competition

**DOI:** 10.1128/mSystems.00108-19

**Published:** 2019-06-11

**Authors:** Alecia N. Septer

**Affiliations:** a Department of Marine Sciences, University of North Carolina, Chapel Hill, North Carolina, USA

**Keywords:** *Aliivibrio*, *Vibrio fischeri*, competition, symbiosis, type VI secretion system

## Abstract

The symbiosis between Euprymna scolopes squid and its bioluminescent bacterial symbiont, Vibrio fischeri, is a valuable model system to study a natural, coevolved host-microbe association. Over the past 30 years, researchers have developed and optimized many experimental methods to study both partners in isolation and during symbiosis. These powerful tools, along with a strong foundational knowledge about the system, position the *Vibrio*-squid symbiosis at the forefront of host-microbe interactions because this system is uniquely suited to investigation of symbiosis from both host and bacterial perspectives. Moreover, the ability to isolate and characterize different strains of V. fischeri has revealed exciting new insights about how different genotypes evolve to compete for a host niche, including deploying interbacterial weapons early during host colonization. This Perspective explores how interbacterial warfare influences the diversity and spatial structure of the symbiotic population, as well as the possible effects that intraspecific competition might have on the host.

## Abstract (precis)

The symbiosis between Euprymna scolopes squid and its bioluminescent bacterial symbiont, Vibrio fischeri, is a valuable model system to study a natural, coevolved host-microbe association. Over the past 30 years, researchers have developed and optimized many experimental methods to study both partners in isolation and during symbiosis.

## PERSPECTIVE

The competitive exclusion principle states that two species with identical niches cannot stably coexist: eventually, one will exclude the other (1). Yet today we know that competing species or strains are often found coexisting in nature. This apparent paradox has inspired researchers and theorists to identify possible mechanisms that allow coexistence of natural competitors. Strategies employed by microorganisms to coexist include sharing physical space but using different metabolic strategies to make a living and establishing “territorial niches” in which competing genotypes deploy interbacterial weapons to spatially separate (2). However, studying the mechanisms and outcomes of competition and coexistence in natural environments is challenging due to their inherent complexity and the dynamic interactions that result from encounters between competitors.

**FIG 1:**
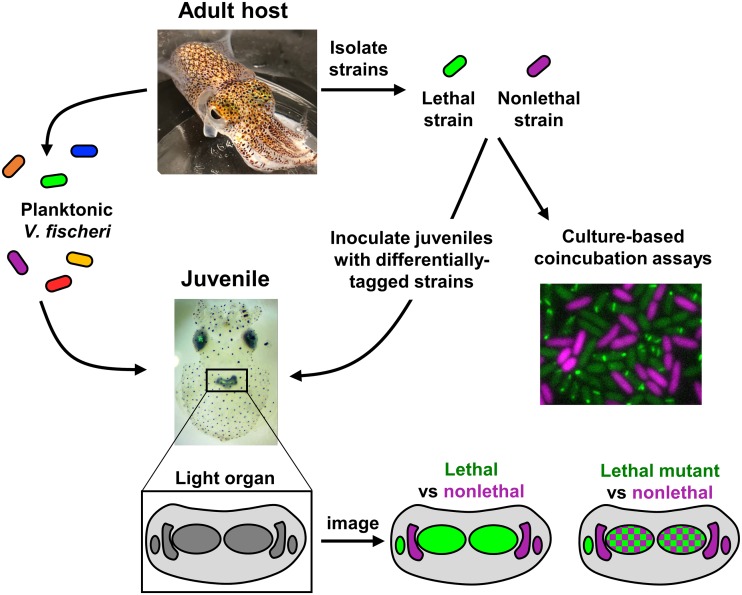
The *Vibrio*-squid symbiosis as a model system for bacterial competition. Adult squid (top left) house multiple strains of V. fischeri bacteria in the light organ. At dawn, ∼90% of the cells are vented into the surrounding seawater, providing a population of colonizers for the next generation of hosts. Juvenile squid (bottom left) hatch without symbionts, which they acquire from the seawater. Using fluorescently tagged isolates, interstrain competition can be visualized both *in vitro* (top right) and *in vivo* (bottom right). Using this approach, we showed previously that lethal (T6SS2^+^) and nonlethal (T6SS2^−^) strains occupy separate crypt spaces but that when T6SS2 is disrupted in the lethal strain, the two strain types can coexist in the same crypt (bottom right) (5). (Images courtesy of Stephanie Smith and Macey Coppinger, reproduced with permission.)

In the *Vibrio*-squid symbiosis (3), multiple strains of a single species (Vibrio fischeri) compete for colonization of the host light organ. Juvenile squid hatch without their symbionts, which they must acquire from the seawater (Fig. 1). Hatchlings become colonized by V. fischeri within hours, and this beneficial infection persists for the lifetime of the squid host. Therefore, the *Vibrio*-squid symbiosis represents a natural and tractable system in which closely related but genetically diverse organisms compete for a single habitat or host niche.

Recent work has shown that competing strains of V. fischeri can coexist in the squid host through a combination of direct and indirect competitive mechanisms. For example, some strains are able to enter and colonize light organ crypts before others (4). Moreover, we recently showed that V. fischeri uses a type VI secretion system (T6SS) to spatially structure the symbiotic population as they establish a mutualistic relationship with their animal host (5). Using multiple, coisolated strains that were taken from wild-caught adult squid, we found that symbiotic V. fischeri contain a strain-specific genomic island that encodes a functional T6SS on chromosome II (T6SS2), which represents a contact-dependent interbacterial weapon (6). Genomic comparisons also revealed that genes encoding the antimicrobial toxins predicted to be translocated by this T6SS from inhibitor to target cells are often strain specific: most strains encode different alleles of toxins, often with no predicted mechanism for their killing abilities. These results suggest that (i) V. fischeri strains rapidly evolve their arsenal of toxins for intraspecific competition; (ii) the mechanism of lethality for these toxins is largely unknown; and (iii) the strain specificity of this weaponry indicates that when different strains come into physical contact with one another, T6SS2-depedent killing results in the elimination of the less fit strain. Thus, light organ isolates are largely incompatible and unable to coexist in the same space, an observation that is consistent with the competitive exclusion principle. Yet we consistently isolate incompatible strains from the same adult light organ (5), suggesting that the paradox of coexisting competitors is also observed in the light organ niche.

One of the strengths of this symbiosis is that the biogeography of the symbiotic population can be mapped using confocal fluorescence microscopy. Two methods include (i) hybridization chain reaction-fluorescent *in situ* hybridization (HCR-FISH) (7) and (ii) colonization of animals with strains that express different fluorescent proteins (FPs). Using the latter approach, several recent studies have revealed how intraspecific competition among V. fischeri strains can impact the diversity and spatial arrangement of strains within the host. Bongrand and Ruby found that strains representing members of a closely related group had the ability to quickly colonize the host and initiate physiological changes in the light organ to discourage subsequent colonization by slower-colonizing competing genotypes (4). Furthermore, Sun et al. reported that certain strain types occupied separate crypts in the light organ and were never observed mixed together (8). Speare et al. determined that this strain separation in the host requires a functional T6SS (Fig. 1) (5). These results suggest that V. fischeri strains have evolved diverse strategies that result in competing genotypes occupying different territorial niches within a single host organ: fast-colonization kinetics can be used to occupy a crypt territory before a competitor arrives, and contact-dependent killing is deployed to exclude a competitor when a crypt is initially cocolonized by two different strains. Together, these findings represent an important step toward understanding how genotypic differences among competing bacteria can shape the host-associated community and underscore the importance of careful strain selection in performing cocolonization assays, as certain strain types can deploy interbacterial weapons.

The *Vibrio*-squid symbiosis is particularly well suited for studying the role of T6SS-dependent competition in a natural system because V. fischeri T6SS2 is active both in the host and in culture. Different strain types can be quantified and visually discriminated within mixed populations using culture-based assays that replicate the competitive interactions observed in the light organ environment (Fig. 1). These assays can be easily modified and scaled up to examine competition under various host-relevant conditions and to identify novel competition factors through high-throughput genetic screens. Moreover, we have engineered a strain in which one of the T6SS2 structural proteins is fused to green fluorescent protein (GFP) (5), allowing direct visualization of T6SS2 sheath assembly (Fig. 1). By using strains expressing a fluorescently tagged T6SS2 component, we can directly observe strain-specific differences in assembly and deployment of this interbacterial weapon when cells are exposed to diverse conditions and competing strain types, as well as to diverse host cells.

This symbiosis also exhibits characteristics ideal for investigating how bacteria evolve mechanisms to compete for the host niche. The squid host can be routinely collected from its natural environment in several bays along the coast of Hawaii (9). Both the squid and seawater in these bays contain an unknown diversity of symbiotic strains that can be selected for through enrichment in juvenile hosts. The ability to isolate many symbiotic genotypes from their natural environment over time provides a unique opportunity to study the evolution of competitive mechanisms among strains as they compete for host colonization sites. Finally, 36 draft genomes are currently available for V. fischeri strains, as well as improved tools for genetic manipulation and transfer of DNA from one strain to another (10), and the host genome has recently been completed and made available (11). Together, these genetic and genomic resources will permit researchers to generate and test new hypotheses about how this intimate host-microbe relationship has coevolved and will reveal new insights into how strain-level diversity among symbionts contributes to the evolution of competitive mechanisms.

Although this system has revealed important findings about how closely related organisms compete for a niche, many issues remain whose resolution will benefit from recently developed techniques. For example, the extent to which the host may be impacted by the secretion of antimicrobial toxins during intraspecific competition remains unknown. Moreover, T6SSs in other bacteria have been shown to inject effectors directly into eukaryotic cells where they can alter host cell physiology (6). Examining interactions between V. fischeri and host cells using established *in vitro* techniques may provide insight into how the T6SS directly or indirectly impacts the host. For example, proteomics data from host hemocytes that have been coincubated with V. fischeri cells (12) may reveal whether host cells respond to nearby intraspecific competition or perhaps are themselves directly injected with T6SS effectors. Furthermore, techniques are available to quantify the host transcriptome (13), which can now be mapped to the squid genome. Such an approach would allow transcriptomics comparisons between animals colonized with competitive or noncompetitive strains, providing a more complete picture of how the host responds to competitive colonization.

Finally, although fierce competition among potential symbionts likely reduces the diversity in the light organ, it is also predicted that competition can increase the stability of a system (2). Future studies could take advantage of recently developed protocols that permit raising juveniles to early adulthood (14) to determine the extent to which interbacterial competition stabilizes the light organ population and its possible effects on host physiology. Such experiments would be particularly insightful if performed with ecologically relevant stressors such as changes in water temperature or exposure to phage (15). The ability to investigate both partners from the initiation of symbiosis through adulthood, in the absence or presence of environmental or biological stressors, has great potential to fill an important gap in our knowledge about how interbacterial warfare impacts host physiology and fitness.
